# Association of ventricular tachycardia burden with 30-day in-hospital mortality in an intensive care unit cohort

**DOI:** 10.1016/j.hroo.2025.08.034

**Published:** 2025-09-01

**Authors:** Jackeline P. Vajta Gomez, Michele M. Pelter, Geoffrey H. Tison, David Mortara, Fabio Badilini, Yumiko Abe-Jones, Sandra Oreper, Margaret C. Fang, Priya A. Prasad

**Affiliations:** 1 Division of Cardiology, Department of Medicine, University of California, San Francisco, Health, San Francisco, California; 2 Center for Biosignal Research, University of California, San Francisco, Health, San Francisco, California; 3 Department of Physiological Nursing, University of California, San Francisco, Health, San Francisco, California; 4 Division of Hospital Medicine, University of California, San Francisco, San Francisco, California

**Keywords:** True ventricular tachycardia, Validated algorithm, Ventricular tachycardia burden, 30-day in-hospital mortality, Intensive care unit

**Key Findings**

▪ True ventricular tachycardia (VT) occurred in 12% of hospitalizations including admission to the intensive care unit.
▪ Patients with a higher VT burden were more likely to have a greater frequency of cardiac comorbidities and other risk factors for poor cardiovascular outcomes.
▪ The odds of 30-day in-hospital mortality increased as the number of true VT events increased (odds ratio 2.46 for 1–2 VT events and 3.70 for ≥3 VT events compared with those with 0 VT events), highlighting a dose-response relationship.

## Introduction

Ventricular tachycardia (VT) is a potentially fatal cardiac rhythm disorder^1^, ^2^, ^3^, ^4^ that most commonly occurs in patients with structural heart disease and occurs in approximately 2% to 13% of patients admitted to the intensive care unit (ICU). VT has been associated with poor outcomes and an increased risk of in-hospital mortality.^5^, ^6^, ^7^, ^8^ Previous studies suggest a dose-response relationship between VT burden and mortality,^3^ which could help identify high-risk patients during continuous electrocardiogram (ECG) monitoring in the ICU—the noninvasive gold standard for detecting VT. However, false VT alarms are extremely common (80%–90%), and studies have failed to establish a consistent methodology for quantifying VT burden in the hospital.^3^^,^^9^, ^10^, ^11^, ^12^ This study aimed to describe VT burden and its association with mortality by leveraging a large consecutive sample of ICU admissions that included expertly annotated true VTs.

## Methods

This was a retrospective observational cohort study among 5320 patients aged ≥18 years with 5679 consecutive ICU admissions to ICUs at the University of California, San Francisco, Medical Center, a tertiary care hospital. Data were collected during a 19-month period (September 2013 to April 2015) and included 3 ICU subtypes (cardiac, medical/surgical, and neurologic). The University of California, San Francisco, Committee on Human Research approved the study (institutional review board 12-09723) with waiver of patient consent, and the research reported adheres to all relevant ethical guidelines, including the Declaration of Helsinki. Patient demographics, 30-day in-hospital mortality, comorbid conditions, Elixhauser risk of mortality scores,^13^ and receipt of arrhythmogenic medications associated with VT or QT prolongation (Supplemental Table 1)^14^ were obtained from the electronic health record. Baseline 12-lead ECG performed in the hospital was used to define the presence of atrial fibrillation/flutter, presence of a pacemaker, bundle branch block, and previous myocardial infarction (Table 1). A research-based closed gateway system was used to collect bedside ECG/physiological monitoring data (GE HealthCare) from the 77 ICU beds during the study period.^5^^,^^10^

**Table 1:** Demographic, clinical characteristics, and outcomes in 5679 hospitalizations including at least 1 ICU admission, stratified by VT burden

Characteristic	0 VT events, n = 5019 (88%)	1–2 VT events, n = 482 (8%)	≥3 VT events, n = 178 (3%)	*P* value
Age at admission, mean (SD)	57.5 (17.5)	60.8 (16.8)	63.0 (16.6)	<.001
Women	2440 (48.6%)	201 (41.7%)	60 (33.7%)	<.001
Elixhauser risk of mortality score at admission, mean (SD)	5.0 (9.1)	8.2 (10.6)	9.8 (11.6)	<.001
Conditions identified by baseline ECG				
Atrial fibrillation or flutter	696 (13.9%)	148 (30.7%)	93 (52.3%)	<.001
Previous VT	49 (1.0%)	19 (3.9%)	27 (15.2%)	<.001
Pacemaker	164 (3.3%)	57 (11.8%)	56 (31.5%)	<.001
Left bundle branch block	83 (1.7%)	32 (6.6%)	27 (15.2%)	<.001
Right bundle branch block	357 (7.1%)	58 (12.0%)	29 (16.3%)	<.001
Previous infarct	551 (11.0%)	143 (29.7%)	67 (37.6%)	<.001
Medical conditions identified by ICD-9/10 code				
Coronary artery bypass graft surgery	145 (2.9%)	27 (5.6%)	19 (10.7%)	<.001
Coronary artery disease	604 (12.0%)	107 (22.2%)	63 (35.4%)	<.001
Heart failure	441 (8.8%)	116 (24.1%)	70 (39.3%)	<.001
Hypertension	1690 (33.7%)	186 (38.6%)	55 (30.9%)	.063
Type 2 diabetes mellitus	823 (16.4%)	103 (21.4%)	49 (27.5%)	<.001
Implantable cardioverter-defibrillator	37 (0.7%)	23 (4.8%)	30 (16.9%)	<.001
ICU type				<.001
Cardiac	752 (15.0%)	133 (27.6%)	80 (44.9%)	
Medical/surgical	1873 (37.3%)	211 (43.8%)	65 (36.5%)	
Neurologic	2394 (47.7%)	138 (28.6%)	33 (18.5%)	
Medications				
Medications associated with QT prolongation	16 (0.32%)	350 (72.6%)	150 (84.3%)	<.001
Medications associated with the risk of VT	206 (4.1%)	115 (23.9%)	75 (42.1%)	<.001
Medications associated with the risk of VT and QT prolongation	3 (0.1%)	105 (21.8%)	80 (44.9%)	<.001
In-hospital mortality (within 30 d)	376 (7.5%)	116 (24.1%)	58 (32.6%)	<.001

ECG = electrocardiogram; ICD-9/10 = International Classification of Diseases, Ninth and Tenth Revisions; ICU = intensive care unit; SD = standard deviation; VT = ventricular tachycardia.

The primary outcome was in-hospital death occurring within 30 days of the first ICU admission during hospitalization. VT burden (primary exposure) was calculated from validated true VT events that were identified using an algorithm that has been previously described and used hospital-based practice standards for VT identification.^5^^,^^15^ VT burden was defined as the total number of true VT events occurring in the ICU during hospitalization and was classified into the following categories: 0 VT events, 1 to 2 VT events, and ≥3 VT events.

### Statistical analysis

Patient data were compared between VT burden categories using standard descriptive statistics. Multivariable logistic regression was used to calculate adjusted odds ratios and 95% confidence intervals (CIs) to examine the association between VT burden categories and 30-day in-hospital mortality. Models were adjusted for patient-level clustering and controlled for demographic and clinical characteristics. Statistical analyses were performed using Stata 14.0 (StataCorp).

## Results

We identified 5679 consecutive hospitalizations with at least 1 ICU admission during the 19-month study period among 5320 unique patients (Table 1). True VT was identified in 660 patients (12%). When comparing the cohort by VT burden category, there were significant differences in age, sex, mortality risk score, cardiac-related comorbidities (except hypertension), presence of an implantable cardioverter-defibrillator, ICU subtype, receipt of medications enhancing VT/QT prolongation, and 30-day in-hospital mortality. Patients with ≥3 VT events were more frequently admitted to the cardiac ICU (44.9%), more often received medications affecting QT prolongation (84.3%), medications that increased VT risk (42.1%), or medications that increase the risk of both QT prolongation and VT (44.9%). They also had a greater 30-day in-hospital mortality (32.6%) than those with 0 or 1 to 2 VT events. Among the 2 groups with VT events, patients in the medical/surgical ICU had the highest proportion of 1 to 2 VT events and patients in the cardiac ICU had the highest proportion of ≥3 VT events.

**Figure 1:**
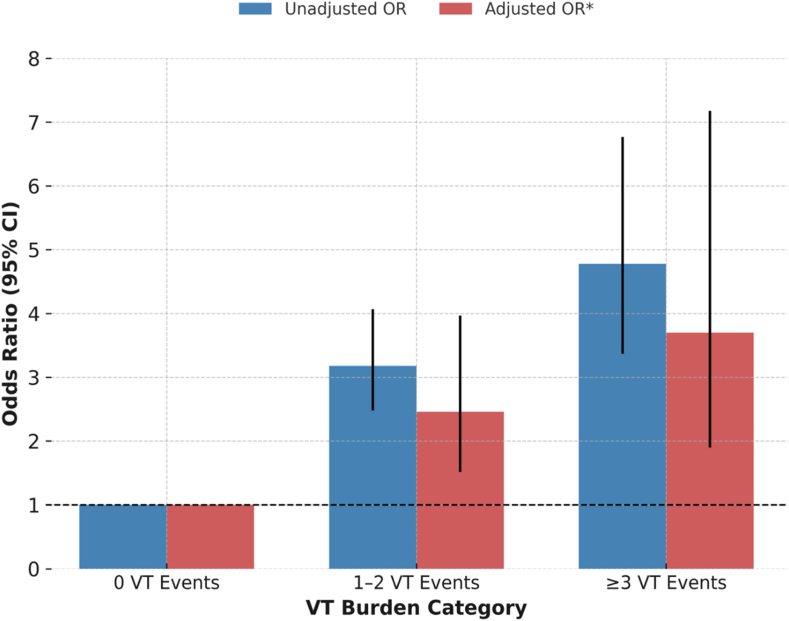
Unadjusted and adjusted ORs for the association between 30-day in-hospital mortality and VT burden category. ∗Adjusted for age, sex, baseline Elixhauser comorbidity index, cardiac-related comorbidities (history of atrial fibrillation, previous VT, pacemaker, left or right bundle branch block, myocardial infarction [MI], previous MI, coronary artery bypass graft surgery, coronary artery disease, heart failure, hypertension, diabetes mellitus, and presence of an implantable cardioverter-defibrillator), medications associated with the risk of VT and QT prolongation (Supplemental Table 1), and ICU subtypes. Models were also adjusted for clustering by patient. OR = odds ratio; VT = ventricular tachycardia.

After adjustment (Figure 1), compared with those with 0 VT events during hospitalization, those with 1 to 2 VT events had 2.46 times the odds of experiencing 30-day in-hospital mortality (95% CI 1.52–3.97) and those with ≥3 VT events had 3.70 times the odds of 30-day in-hospital mortality (95% CI 1.90–7.18).

## Discussion

Our study is one of the first to quantify VT burden using expertly annotated true VT events and measure the association between hospital-based VT burden and 30-day in-hospital mortality in an adult ICU population. Our study demonstrated that the odds of 30-day in-hospital mortality increased as the number of true VT events increased, even after adjustment for potential confounders. Our findings suggest a dose-response relationship between VT burden and 30-day in-hospital mortality, corroborating and adding to other nonhospital-based studies.^3^^,^^11^^,^^12^

Although the burden of atrial fibrillation and other arrhythmias has been previously defined for risk stratification,^16^^,^^17^ a similar VT burden definition is currently lacking. Although the occurrence of a single VT may be critical and VT burden may not be considered clinically relevant, previous work from our group in a smaller cohort demonstrated large variations in VT occurrence rates per patient (1–20 events), VT duration (seconds), and maximal heart rate per VT event, suggesting that not all VT is the same in ICU patients.^9^ The current study aimed to define VT burden in a larger ICU sample using the total number of true VT events, but other VT features need to be examined further. For example, classification of VT by duration (nonsustained vs sustained [>30 seconds]), QRS direction (positive vs negative), QRS width, and hemodynamic impact (blood pressure and oxygen saturation) would be important next steps, which our group is undertaking.^2^ We hypothesize that this would add precision to current hospital-based ECG monitoring systems and help identify patients at the highest risk of untoward outcomes.

### Limitations

We did not annotate other key VT features (heart rate, duration, QRS direction, hemodynamic response), although our group is assessing these now. We also did not distinguish monomorphic from polymorphic VT, which may carry different mortality risks, although our dataset contained few polymorphic cases. Finally, we did not separate actionable VTs (prompting medication or electrolyte changes, defibrillation, or response to cardiac arrest) from nonactionable VTs, which could help clarify VT burden and optimize alarm settings to reduce staff fatigue. Despite these limitations, our study demonstrates a VT dose–response relationship associated with increased 30-day in-hospital mortality.

## Conclusion

VT burden, defined as the total number of true VT events occurring in the ICU during hospitalization, is a predictor of 30-day in-hospital mortality. There is a dose-response relationship with a higher risk of mortality in patients with ≥3 VT events. Future work should be done to create more specific definitions of VT burden to accurately stratify patients at the greatest risk of poor outcomes who may benefit from early and aggressive targeted interventions.

## Declaration of generative AI and AI-assisted technologies in the writing process

During the preparation of this work, the authors used ChatGPT to help generate Figure 1. After using this tool/service, the authors reviewed and edited the content as needed and take responsibility for the content of the publication.

## Disclosures

Dr Prasad reports personal fees from Epi Excellence, LLC, outside the submitted work. This study was funded by the University of California, San Francisco, School of Nursing Lipps Research Fund (Pelter as the principal investigator). Dr Pelter was supported by an R01 from the National Heart, Lung, and Blood Institute (HL-167975, Pelter as the principal investigator).
