# Supplementary material for: Association of ventricular tachycardia burden with 30-day in-hospital mortality in an intensive care unit cohort
Source: Heart Rhythm O2. 2025 Sep 1;6(11):1769–72. doi: 10.1016/j.hroo.2025.08.034 (PMC12675054; doi:10.1016/j.hroo.2025.08.034)
Supplement: Supplementary Table 1 [file mmc1.docx]

**Supplemental Table 1.** Medications that increase the risk of ventricular tachycardia (VT) and/or cause QT prolongation(leading to VT). [^14^](https://sciwheel.com/work/citation?ids=9704568&pre=&suf=&sa=0&dbf=0)

| **Drug Type** | **Drug Name** | **Associated Risk** |
| --- | --- | --- |
| Antiarrhythmic | Adenosine | VT |
| Antiarrhythmic | Amiodarone | VT/QT |
| Antibiotic | Azithromycin | QT |
| Anesthetic | Bupivacaine | VT |
| Antidepressant | Bupropion | VT |
| Antipsychotic | Chlorpromazine | VT/QT |
| Antibiotic | Ciprofloxacin | QT |
| Antidepressant | Citalopram | VT/QT |
| Antibiotic | Clarithromycin | QT |
| Antidepressant | Desipramine | VT |
| Inotrope | Digoxin | VT |
| Antiplatelet | Dipyridamole | VT |
| Inotrope | Dobutamine | VT |
| Cholinesterase inhibitor | Donepezil | QT |
| Antibiotic | Erythromycin | QT |
| Antidepressant | Escitalopram | QT |
| Antiarrhythmic | Flecainide | VT/QT |
| Antifungal | Fluconazole | QT |
| Antipsychotic | Haloperidol | QT |
| Antimalarial | Hydroxychloroquine | QT |
| Antidepressant | Imipramine | VT |
| Anticonvulsant | Lacosamide | VT |
| Antibiotic | Levofloxacin | QT |
| Antimanic | Lithium | VT |
| Opioid agonist | Methadone | QT |
| Inotrope | Milrinone | VT |
| Antibiotic | Moxifloxacin | QT |
| Antiemetic | Ondansetron | QT |
| Antifungal | Pentamidine | QT |
| Antiarrhythmic | Procainamide | VT/QT |
| Antiarrhythmic | Propafenone | VT |
| Anesthetic | Propofol | QT |
| Antiarrhythmic | Quinidine | QT |
| Anesthetic | Ropivacaine | VT |
| Antiarrhythmic | Sotalol | VT/QT |
| Phosphodiesterase | Theophylline | VT |
| Antidepressant | Venlafaxine | VT |
